# On the Performance of Underlay Device-to-Device Communications

**DOI:** 10.3390/s22041456

**Published:** 2022-02-14

**Authors:** Tan Nhat Nguyen, Van Son Nguyen, Hoai Giang Nguyen, Lam Thanh Tu, Trinh Van Chien, Tien Hoa Nguyen

**Affiliations:** 1 Communication and Signal Processing Research Group, Faculty of Electrical and Electronics Engineering, Ton Duc Thang University, Ho Chi Minh City 756000, Vietnam; nguyennhattan@tdtu.edu.vn; 2 Faculty of Electronics Telecommunication & Information Technology, Hanoi Open University, Hanoi 100000, Vietnam; sonnv@hou.edu.vn (V.S.N.); giangnh@hou.edu.vn (H.G.N.); 3 Institute XLIM, University of Poitiers, 86073 Poitiers, France; lam.thanh.tu@univ-poitiers.fr; 4 Interdisciplinary Centre for Security, Reliability and Trust, University of Luxembourg (SnT), 1855 Luxembourg, Luxembourg; vanchien.trinh@uni.lu; 5 School of Electronics and Telecommunications, Hanoi University of Science and Technology, Hanoi 100000, Vietnam

**Keywords:** average rate, amount of fading, device-to-device, outage probability, performance analysis

## Abstract

This paper comprehensively investigates the performance of the D2D underlaying cellular networks where D2D communications are operated concurrently with cellular networks provided that the aggregate interference measured on licensed users is strictly guaranteed. In particular, we derive the outage probability (OP), the average rate, and the amount of fading (AoF) of the D2D networks in closed-form expressions under three distinct power allocation schemes, i.e., the path-loss-based, equal, and random allocation schemes. It is noted that the considered networks take into consideration the impact of the intra-D2D networks, the inter-interference from the cellular networks and background noise, thus involving many random variables and leading to a complicated mathematical framework. Moreover, we also reveal the behavior of the OP with respect to the transmit power based on the rigorous mathematical frameworks rather than the computer-based simulation results. The derived framework shows that increasing the transmit power is beneficial for the OP of the D2D users. Regarding the cellular networks, the coverage probability (Pcov) of the cellular users is computed in closed-form expression too. Monte Carlo simulations are given to verify the accuracy of the proposed mathematical frameworks. Our findings illustrate that the power allocation method based on prior path-loss information outperforms the other methods in the average sum rate.

## 1. Introduction

Device-to-device (D2D) communication technology is one of the most effective solutions to ameliorate both spectral and energy efficiency [1,2]. Specifically, D2D allows two proximity users to communicate without utilizing the infrastructure of the cellular networks, hence easing the pressure on enhancing the cellular network owing to the exponential growth of radio-connected devices and their power and bandwidth intensive applications [3]. Additionally, by shortening the transmission distance, the quality-of-service (QoS) in terms of the end-to-end (e2e) delay, both the reliability and the high data rate, are guaranteed [4]. Compared with other short-range wireless transmission technologies such as WiFi Direct, Bluetooth, and ultra-wide bandwidth, D2D is preferable due to the inheritance of well-standardized, well-structured cellular networks. Furthermore, it directly accesses the licensed spectrum, enjoying a high data rate and less interference. Another notable benefit of D2D communications permits us to offload cellular traffic, thus avoiding congestion in core networks [5]. D2D communications have therefore gained attention from both academia and industry. In the next section, the state-of-the-art of the D2D networks itself, as well as in combination with other techniques, is provided.

## 2. State-of-the-Art

One advantage of D2D communications is that it can easily combine with other advanced techniques to further enhance the performance of wireless communications systems. In particular, the work in [6,7,8] investigated the performance of massive multiple-input multiple-output (MIMO) with D2D-enabled communications. It derived the lower bound of the capacity of cellular users by assuming that all cells share the same set of pilots [7]. He et al. [8], proposed a dynamic power control policy for mitigating the inter-interference in underlay D2D massive MIMO cellular networks. The work in [9,10] addressed the performance of the D2D-enabled relaying networks. More specifically, Kamal et al. in [10] studied bit error rate (BER) and spectral efficiency where base stations (BSs) applied spatial modulation (SM). The joint optimization of the relay selection, spectrum allocation, and power control was investigated in dual-hop communications by employing the matching theory in [9]. Their findings unveiled that the performance in the presence of a relay was far better than the case without relays. Adeel et al. [11], on the other hand, investigated the security problem of the D2D relay-assisted networks. In particular, their proposed scheme, which was suitable for any infrastructure, was significantly enhanced the security of any D2D settings. The works in [12,13,14] applied tools from stochastic geometry (SG) in order to better capture the randomness of both the D2D and the cellular users. More specifically, the closed-form expressions of average queue length, average throughput, average delay, and dropping probability were evaluated in [12]. On the other hand, authors in [13] derived coverage probability (Pcov) of the cellular users. The performance of the D2D-assisted mmWave communications was provided in [15,16,17,18]. In particular, the offloading gain of the D2D mmWave communications was studied in both half- and full-duplex in [18], while [15] discussed the potential of D2D-assisted mmWave communications. The combination of the D2D and Long-Range (LoRa) networks was studied in [19,20], while the throughput-outage analysis of D2D caching networks was addressed in [21]. The work in [22], meanwhile, addressed the sum rate of the NOMA-based cellular networks with imperfect self-interference cancellation. This work, nonetheless, computed the sum rate via numerical results in place of the rigorous mathematical framework. Authors in [23] studied the outage probability of the cognitive radio D2D transmission. The amount of fading (AoF) of the D2D networks was investigated in [24,25], however, it was derived based on the Fox-H function, which is not stable and mathematically intractable. Mankar et al. [26] derived the system throughput under the assumption of interference limitation, while the subchannel and power allocation were addressed in [27,28].

The performance of the D2D networks itself was studied widely in [29,30,31,32,33,34,35,36,37,38]. The work in [29] investigated the fairness scheduling policies in multi-user diversity D2D cellular networks. In particular, two fairness scheduling policies were considered in that work, i.e., cellular fairness scheduling (CFS) and D2D fairness scheduling (DFS). The outcomes illustrated that the DFS scheme is far better than the CFS scheme in terms of the effective rate and the access probability. A heuristic algorithm for proportional fair scheduling (PFS) was proposed in [30] to maximize the logarithm sum of average rate. The proposed algorithm achieved a high sum of average rate while maintaining low computational complexity. Bo et al. devised a social-aware virtual medium access control (SV-MAC) to integrate virtualization and social-awareness to D2D cellular networks [31]. Under the proposed protocol, the considered network is unified in a single protocol stack, giving a common abstraction for end-users and mobile network operators (MNOs) to share their network resources. The results reveal that the proposed protocol outperforms both the conventional networks and the social-aware no virtualization model in terms of the energy efficiency. The resource allocation of the uplink heterogeneous cellular networks was addressed in [32], where the Kuhn–Munkres (KM) algorithm was employed to maximize the total capacity of D2D networks. Both power allocation and link scheduling in D2D-assisted wireless caching networks were addressed in [33]. The emergency route selection (ERS) of D2D cellular networks during an urban terrorist attack were studied. More precisely, different wireless routing algorithms, namely shortest-path-routing (SPR), interference-aware-routing (IAR), and broadcast-routing (BR) were studied in [36]. Simulation results depicted that the IAR was the best routing scheme, the SPR scheme was the second best, and BR was the worst. An adaptive IAR scheme was also proposed in [37] which improved the network capacity by over 50%. A novel routing algorithm that can achieve the highest trusted connectivity probability (TCP) of any pair of D2D devices was proposed in [38]. Computer-based results presented that the proposed algorithm attained almost the same performance as the exhaustive search.

The most closely related research works available in the literature were [39,40,41,42,43]. However, authors in [39] merely studied the outage probability by assuming that the system was interference-limited, thus ignoring the impact of background noise. However, it is obvious that under the sparsely loaded scenario, the D2D transmission is dominated by background noise. Additionally, from the mathematical framework point of view, without the presence of noise, the complexity of the derived framework significantly reduces. Furthermore, they merely considered the equal power allocation rather than optimizing the transmit power in order to substantially enhance the system performance. The average rate and AoF are considered the most important metrics in wireless communications; nevertheless, they did not study these essential metrics. Regarding [40], the authors addressed the channel allocation problem in the D2D networks. Although both interference and noise were taken into consideration in their work, they did not compute the outage probability in the closed-form expression. In fact, a numerical algorithm was proposed to solve the considered problem; however, this did not allow one to study the trend/behavior of the system metric with respect to an important metric, e.g., the transmit power, via the rigorous mathematical framework. Moreover, as in [39], neither the average rate or amount of fading are taken into account. As for [41], the author also investigated the OP. Nevertheless, it considered a simplified scenario that there is only a pair of D2D users; thus, no intra-D2D interference is taken into consideration. Additionally, they also ignored the background noise when addressing the power control problem that obviously simplifies the framework. Finally, as in [39,40] both average rate and amount of fading are skipped in [41]. Authors in [42] studied the outage probability and the moment generating function (MGF) of the interference in millimeter-wave with the absence of noise. This work, nonetheless, did not study the impact of the transmit power on the performance of the outage probability and the average rate and the amount of fading as in [39,40,41]. Similarly, the work in [43] likewise skipped the impact of noise, nor was the power allocation considered.

In the present paper, different from the above-mentioned works, we study not only the outage probability but also the average rate and amount of fading of D2D networks and the coverage probability of the cellular networks. It is obvious that the derivation of the average rate is more challenging than the OP due to the additional integration from the probability density function multiplied with the logarithm function. Additionally, we take into consideration both the interference and noise. It is emphasized that considering both the interference and noise together will typically lead to an intractable mathematical framework. We, however, are able to derive all metrics in the closed-form expressions. In addition, we consider different power allocation schemes rather than only the equal power allocation as in other works. The main contributions and novelties are summarized as follows:

- To the best of authors knowledge, this is the first work that computes the outage probability, average rate, and amount of fading of the D2D networks under the impact of interference from D2D and cellular networks, as well as the background noise in closed-form expressions with three distinct power allocation schemes, namely, the equal, random, and path-loss based schemes.
- We derive the coverage probability of the cellular users under the aggregate interference of D2D transmission.
- The impact of the total transmit power of D2D networks on the performance of the OP is explicitly studied.
- Monte Carlo simulation results are presented to verify the accuracy of the proposed mathematical framework as well as to highlight the behavior of these metrics with respect to system parameters.

The remainder of this paper is organized as follows: The system model is given in Section 3. The derivation of key performance metrics, e.g., the OP, the average rate and the AoF, is provided in Section 4. Numerical results are presented in Section 5. Section 6 concludes the paper. The list of notations and acronyms are given in Table 1.

## 3. System Model

In this section, a detailed system model is provided. In particular, we discuss the procedure operation of the whole networks, the large-scale path-loss, and small-scale fading. Additionally, three distinct power allocation schemes, i.e., the equal, random, and path-loss-based schemes, are provided. Then, based on the received signals, the signal-to-interference-plus-noise (SINR) at both D2D receivers and base station are formulated.

**Figure 1:**
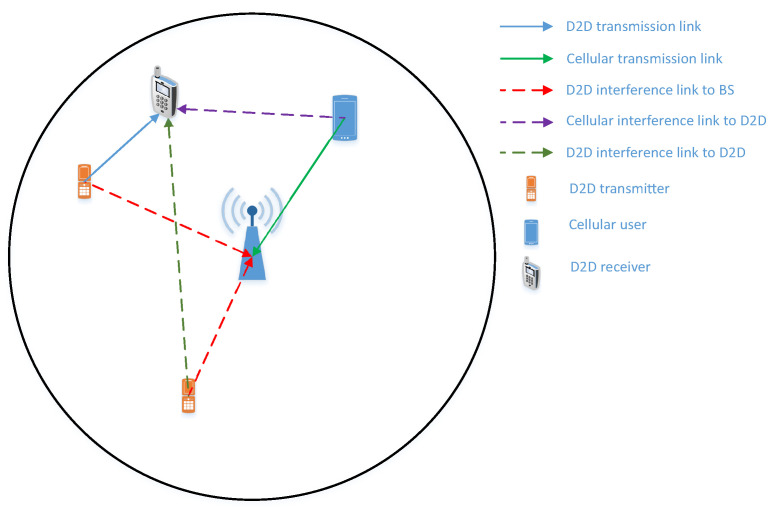
System model of D2D communication underlaying cellular network.

Let us consider a single cell uplink cellular networks with *N* pairs of device-to-device users and cellular users, as shown in Figure 1. We assume that all nodes, i.e., the cellular and D2D users, are located inside the coverage area of the base station (BS) which is a disk with the center at the BS and *O* radius. It is noted that there is at most one node at a specific location under the considered networks. The D2D users are operated under the underlay protocol which permits them to continuously access the licensed spectrum provided that the aggregate interference at the base station is less than a predefined threshold [44]. The whole transmission takes place in one resource block (RB), where the D2D transmitters send their information to their dedicated D2D receiver (Here, an RB can be a time resource or a frequency resource [45]).

Meanwhile, the cellular user also transmits its signals to the base station. (The present paper considers an RB (one time-lot) scenario. However, if the multiple RBs scenario is adopted, the cellular user, of course, is able to access all RBs. As for the D2D users, they can also access all RBs, provided that the interference created by the D2D networks measured on the cellular users is strictly guaranteed. Additionally, the derived mathematical framework in the present paper can straightforwardly apply to the multiple RBs scenario where all RBs have at least one active cellular user.) As a result, D2D receivers are subjected to interference from both D2D networks and cellular networks. The BS, however, only experiences interference from the D2D networks due to the orthogonal resource allocation at the cellular networks such as time-division multiple access (TDMA) and/or frequency-division multiple access (FDMA). As all transmissions take place simultaneously, a power control scheme is needed at the D2D networks in order to guarantee the interference and quality of service (QoS) at the cellular networks, as well as to enhance the performance of the D2D networks. Three distinct power allocation schemes are considered in the present paper and are given in detail in Section 3.2.

### 3.1. Channel Modeling

Let us denote $h_{i,j}$ the channel coefficient between the transmitter $j\in \left\{dt,c\right\}$ and the receiver $i\in \left\{dr,b\right\}$; where dt, dr,b, and *c* denote the D2D transmitter, the D2D receiver, the base station, and the cellular user, respectively. Here, we assume that $h_{i,j}$ follows complex Gaussian random variable with zero mean and $\Omega_{i,j}$ variance. The absolute of $h_{i,j}$, i.e., $\left|h_{i,j}\right|$ is then followed by the Rayleigh distribution. In the present paper, Rayleigh fading is adopted as it is considered the worst fading. As a result, the system performance under the impact of Rayleigh fading is the lower bound compared with other distributions such as Rician, Nakagami-*m*, and Weibull distributions, etc. $\Omega_{i,j}$ is also the large-scale path-loss from transmitter *j* to receiver *i*. To be more precise, $\Omega_{i,j}$ is formulated as [46,47]

(1) $$\begin{array}{c} \Omega_{i,j}=K_{0}d_{i,j}^{\beta }, \end{array}$$

where $K_{0}={\left(4\pi /v\right)}^{2}$ and β are the path-loss constant and the path-loss exponent; $v=c_{0}/f_{c}$ is the wavelength; $c_{0}$ is the speed of light measured in m/s; and $f_{c}$ is the carrier frequency. We notice that the considered unbounded path-loss modeling can be straightforwardly extended to the bounded path-loss model to overcome the singularity issue [48].

### 3.2. Power Allocation in D2D Networks

In this paper, we adopt three distinct power allocation schemes in the D2D networks comprising the equal, random, and path-loss-based schemes [49]. The detailed definition of these schemes are given below.

#### 3.2.1. Equal Power Allocation

Under this scheme, the transmit power of all D2D transmitters is exactly the same and is computed as [6]

(2) $$\begin{array}{c} P_{D_{k}}^{equ}=\frac{P_{\mathrm{tot}}}{N},k\in \left\{1,\ldots ,N\right\}, \end{array}$$

where $P_{\mathrm{tot}}$ is the total transmit power of the D2D networks and $P_{D_{k}}$ is the transmit power of the *k*-th transmitter.

#### 3.2.2. Random Power Allocation

Let us denote $P_{D_{k}}^{ran}$ the transmit power of the *k*-th D2D transmitter under the random power allocation, its value is randomly assigned and given as [6]

(3) $$\begin{array}{c} P_{D_{k}}^{ran}=\alpha_{k}P_{\mathrm{tot}},\;\forall k,\;\alpha_{k}\in \left(0,1\right),\mathrm{and}\sum_{k=1}^{N}\alpha_{k}=1, \end{array}$$

where $\alpha_{k}$ is a random number generated by a uniform distribution.

#### 3.2.3. Path-Loss-Based Power Allocation

Under the path-loss-based allocation scheme, the transmit power of the D2D transmitter is allocated based on the large-scale path-loss to its intended receiver. More precisely, the D2D pair, having larger path-loss, will be allocated a higher transmit power to complement the long transmission distance. The transmit power of the *k*-th D2D transmitter under this method (denoted by $P_{D_{k}}^{pl}$) is then computed as

(4) $$\begin{array}{c} P_{D_{k}}^{pl}=\frac{\Omega_{dr_{k},dt_{k}}}{\sum_{i=1}^{N}\Omega_{dr_{i},dt_{i}}}P_{\mathrm{tot}},\forall k. \end{array}$$

Direct inspection of (2)–(4) makes it apparent that the transmit power of the *k*-th D2D transmitter can be represented as [6]

(5) $$\begin{array}{c} P_{D_{k}}^{s}=\omega_{k}^{s}P_{\mathrm{tot}},\;\forall k,\;s\in \left\{equ,ran,pl\right\}, \end{array}$$

where $\omega_{k}^{equ}=\frac{1}{N}$, $\omega_{k}^{ran}=\alpha_{k}$, and $\omega_{k}^{pl}=\frac{\Omega_{dr_{k},dt_{k}}}{\sum_{i=1}^{N}\Omega_{dr_{i},dt_{i}}}$.

It is noted that both the centralized and decentralized power allocation can be adopted in wireless communications. For the equal power allocation, it is easy to implement in a distributed manner. As we consider a single-cell D2D underlaying cellular networks, thus the two remaining power allocation schemes can be effectively centralized implemented at the core network; then, the power will be assigned to each D2D transmitter.

### 3.3. Signal-to-Interference-Plus-Noise Ratio (SINR) at D2D Receivers and Base Station

The received signal at the *k*-th D2D receiver and the BS under the $s\in \left\{equ,ran,pl\right\}$ power allocation scheme are formulated as [50,51]

(6) $$\begin{array}{cc} y_{D_{k}}= & \underset{\mathrm{Useful}\;\mathrm{signal}}{\underset{︸}{\sqrt{P_{D_{k}}^{s}}h_{dr_{k},dt_{k}}x_{D_{k}}}}+\underset{D2D\;\mathrm{interference}}{\underset{︸}{\sum_{i=1,i\neq k}^{N}\sqrt{P_{D_{i}}^{s}}h_{dr_{k},dt_{i}}x_{D_{i}}}}+\underset{\mathrm{Cellular}\;\mathrm{interference}}{\underset{︸}{\sqrt{P_{C}}h_{dr_{k},c}x_{C}}}+n_{D_{k}} \end{array}$$

(7) $$\begin{array}{cc} y_{b}= & \underset{\mathrm{Useful}\;\mathrm{signal}}{\underset{︸}{\sqrt{P_{C}}h_{b,c}x_{C}}}+\underset{D2D\;\mathrm{interference}}{\underset{︸}{\sum_{i=1}^{N}\sqrt{P_{D_{i}}^{s}}h_{b,dt_{i}}x_{D_{i}}}}+n_{b}. \end{array}$$

Here $x_{D_{k}}$, $k\in \left\{1,\ldots ,N\right\}$ and $x_{C}$ with $E\left\{{\left|x_{D_{k}}\right|}^{2}\right\}=E\left\{{\left|x_{C}\right|}^{2}\right\}=1$ are the transmitted signal of the *k*-th D2D transmitter and cellular user, respectively; $E\left\{.\right\}$ is the expectation operator; and $n_{D_{k}}$ and $n_{b}$ are the additive white Gaussian noise (AWGN) at the *k*-th D2D receiver and at the BS, respectively. Looking at (6) and (7), we ascertain that the D2D receiver is subjected to interference from both the D2D and the cellular networks, while the BS is only affected by the D2D interference. The signal-to-interference-plus-noise-ratio (SINR) of the *k*-th D2D receiver and the BS under the *s* scheme (denoted by $\Xi_{k}^{s}$ and $\Delta^{s}$) is then evaluated as [52]

(8) $$\begin{array}{cc} \Xi_{k}^{s}= & \frac{P_{D_{k}}^{s}{\left|h_{dr_{k},dt_{k}}\right|}^{2}}{\sum_{i=1,i\neq k}^{N}P_{D_{i}}^{s}{\left|h_{dr_{k},dt_{i}}\right|}^{2}+P_{C}{\left|h_{dr_{k},c}\right|}^{2}+\sigma_{k}^{2}}, \end{array}$$

(9) $$\begin{array}{cc} \Delta^{s}= & \frac{P_{C}{\left|h_{b,c}\right|}^{2}}{\sum_{i=1}^{N}P_{D_{i}}^{s}{\left|h_{b,dt_{i}}\right|}^{2}+\sigma_{b}^{2}}, \end{array}$$

where $\sigma_{k}^{2}$, $k\in \left\{1,\ldots ,N\right\}$ and $\sigma_{b}^{2}$ are noise variance of the *k*-th D2D receiver and the base station, respectively, and are calculated as $\sigma_{D_{k}}^{2}=\sigma_{b}^{2}=-174+\mathrm{NF}+10\log_{10}\left(\mathrm{Bw}\right),\forall k$ [53]; NF is the receiver noise figure in dB; and Bw is the transmission bandwidth in Hz.

## 4. Performance Analysis

In the present paper, we investigate the performance of both the cellular networks and D2D networks under three distinguished power allocation schemes. In particular, we derive the outage probability, the average rate, and the amount of fading of the D2D receiver. Regarding the cellular networks, we derive the coverage probability under the impact of the aggregate interference from all D2D transmissions. Before going to derive these metrics, we first present Theorem 1, which is useful for later computation.

**Theorem** **1.** *Let us denote $X_{1},X_{2},\ldots ,X_{N}$ as a set of independent non-identical distributed (i.n.i.d) exponential random variables (RVs) with corresponding parameters $\Omega_{1},\Omega_{2},\ldots ,\Omega_{N}$, the moment generating function (MGF) of sum of N RVs, i.e., $Y=\sum_{i=1}^{N}X_{i}$, denoted by $M_{Y}\left(s\right)$ is calculated as [54]*

(10) $$\begin{array}{c} M_{Y}\left(s\right)=\prod_{i=1}^{N}\frac{1}{1+s\Omega_{i}}. \end{array}$$

**Proof** **of Theorem 1.** Let us denote the joint probability density function (PDF) of *N* exponential RVs as $f_{X_{1},\ldots ,X_{N}}\left(x_{1},\ldots ,x_{N}\right)$, the MGF of the sum of *N* RVs, e.g., $Y=\sum_{k=1}^{N}X_{k}$ is then computed as [14]

(11) $$\begin{array}{cc} M_{Y}\left(s\right)=E\left\{\exp\left(-sy\right)\right\}= & \int_{x_{1}=0}^{\infty }\ldots \int_{x_{N}=0}^{\infty }\exp\left(-s\sum_{k=1}^{N}x_{k}\right)f_{X_{1},\ldots ,X_{N}}\left(x_{1},\ldots ,x_{N}\right)dx_{1}...dx_{N}\\ \overset{\left(a\right)}{=} & \prod_{k=1}^{N}\int_{x_{k}=0}^{\infty }\exp\left(-sx_{k}\right)f_{X_{k}}\left(x_{k}\right)dx_{k}\\ \overset{\left(b\right)}{=} & \prod_{k=1}^{N}\frac{1}{\Omega_{k}}\int_{x_{k}=0}^{\infty }\exp\left(-sx_{k}-\frac{x_{k}}{\Omega_{k}}\right)dx_{k}\\ = & \prod_{i=1}^{N}\frac{1}{1+s\Omega_{i}}, \end{array}$$

where $\left(a\right)$ is held due to the independent property of these RVs; substituting the PDF of the exponential RV we obtain $\left(b\right)$. Q.E.D. □

Having obtained the MGF of the sum of the arbitrary exponential RVs, we are now ready to compute the outage probability.

### 4.1. OP of the D2D User

Outage probability is referred to the probability that the end-to-end SINR is below a predefined threshold [55]. More precisely, let us denote ${\mathrm{OP}}_{k}^{s}\left(\gamma \right)$ as the outage probability of the *k*-th D2D receiver under the $s\in \left\{equ,ran,pl\right\}$ scheme, it can be computed as [56]

(12) $$\begin{array}{cc} {\mathrm{OP}}_{k}^{s}\left(\gamma \right)= & \mathrm{Pr}\left(\Xi_{k}^{s}=\frac{P_{D_{k}}^{s}{\left|h_{dr_{k},dt_{k}}\right|}^{2}}{\sum_{i=1,i\neq k}^{N}P_{D_{i}}^{s}{\left|h_{dr_{k},dt_{i}}\right|}^{2}+P_{C}{\left|h_{dr_{k},c}\right|}^{2}+\sigma_{k}^{2}}<\gamma \right)\\ = & \mathrm{Pr}\left(\frac{P_{D_{k}}^{s}{\left|h_{dr_{k},dt_{k}}\right|}^{2}}{I_{k}+P_{C}{\left|h_{dr_{k},c}\right|}^{2}+\sigma_{k}^{2}}<\gamma \right)\\ = & \mathrm{Pr}\left({\left|h_{dr_{k},dt_{k}}\right|}^{2}<\frac{\gamma }{P_{D_{k}}^{s}}\left(I_{k}+P_{C}{\left|h_{dr_{k},c}\right|}^{2}+\sigma_{k}^{2}\right)\right)\\ = & \int_{i=0}^{\infty }\int_{x=0}^{\infty }\left(1-\exp\left(-\frac{\gamma \sigma_{k}^{2}}{P_{D,k}^{s}\Omega_{dr_{k},dt_{k}}}-\frac{P_{C}\gamma }{P_{D_{k}}^{s}\Omega_{dr_{k},dt_{k}}}x-\frac{\gamma }{P_{D_{k}}^{s}\Omega_{dr_{k},dt_{k}}}i\right)\right)\\ \; & \times f_{I_{k}}\left(i\right)f_{{\left|h_{dr_{k},c}\right|}^{2}}\left(x\right)didx\\ = & 1-\exp\left(-\frac{\gamma \sigma_{k}^{2}}{P_{D_{k}}^{s}\Omega_{dr_{k},dt_{k}}}\right)\int_{i=0}^{\infty }\int_{x=0}^{\infty }\exp\left(-\frac{P_{C}\gamma }{P_{D_{k}}^{s}\Omega_{dr_{k},dt_{k}}}x\right)\exp\left(-\frac{\gamma }{P_{D_{k}}^{s}\Omega_{dr_{k},dt_{k}}}i\right)f_{I_{k}}\left(i\right)\\ \; & \times f_{{\left|h_{dr_{k},c}\right|}^{2}}\left(x\right)didx\\ \overset{\left(a\right)}{=} & 1-\exp\left(-\frac{\gamma \sigma_{k}^{2}}{P_{D_{k}}^{s}\Omega_{dr_{k},dt_{k}}}\right)M_{I_{k}}\left(\frac{\gamma }{P_{D_{k}}^{s}\Omega_{dr_{k},dt_{k}}}\right)M_{{\left|h_{dr_{k},c}\right|}^{2}}\left(\frac{P_{C}\gamma }{P_{D_{k}}^{s}\Omega_{dr_{k},dt_{k}}}\right)\\ \overset{\left(b\right)}{=} & 1-\exp\left(-\frac{\gamma \sigma_{k}^{2}}{P_{D_{k}}^{s}\Omega_{dr_{k},dt_{k}}}\right)\left(\prod_{i=1,i\neq k}^{N}\frac{1}{1+\frac{\gamma }{P_{D_{k}}^{s}\Omega_{dr_{k},dt_{k}}}P_{D_{i}}^{s}\Omega_{dr_{k},dt_{i}}}\right)\frac{1}{1+\frac{P_{C}\gamma \Omega_{dr_{k},c}}{P_{D_{k}}^{s}\Omega_{dr_{k},dt_{k}}}}\\ = & 1-\exp\left(-\frac{\gamma \sigma_{k}^{2}}{P_{D_{k}}^{s}\Omega_{dr_{k},dt_{k}}}\right)\frac{A_{k,c}^{s}}{A_{k,c}^{s}+\gamma }\left(\prod_{i=1,i\neq k}^{N}\frac{B_{k,i}^{s}}{B_{k,i}^{s}+\gamma }\right), \end{array}$$

where $A_{k,c}^{s}=\frac{P_{D_{k}}^{s}\Omega_{dr_{k},dt_{k}}}{P_{C}\Omega_{dr_{k},c}}$, $B_{k,i}^{s}=\frac{P_{D_{k}}^{s}\Omega_{dr_{k},dt_{k}}}{P_{D_{i}}^{s}\Omega_{dr_{k},dt_{i}}}=\frac{\omega_{k}^{s}\Omega_{dr_{k},dt_{k}}}{\omega_{i}^{s}\Omega_{dr_{k},dt_{i}}}$, $\gamma =2^{R}-1$ where *R* is the target rate in bits/s/Hz; $\mathrm{Pr}\left(.\right)$ is the probability operator; $I_{k}=\sum_{i=1,i\neq k}^{N}P_{D_{i}}^{s}{\left|h_{dr_{k},dt_{i}}\right|}^{2}$ is the aggregate interference at the *k*-th D2D receiver creating by other D2D transmitters. $\left(a\right)$ is obtained by using the definition of the MGF function; $\left(b\right)$ is held with the help of Theorem 1. In the following, we compute the average rate of the D2D networks.

### 4.2. Average Rate of D2D User

Let us denote ${\bar{R}}_{k}^{s}$ as the average rate of the *k*-th D2D user under the *s* scheme and is computed as follows [52,57]:

(13) $$\begin{array}{cc} \; & {\bar{R}}_{k}^{s}=E\left\{\log_{2}\left(1+\Xi_{k}^{s}\right)\right\}=\int_{0}^{\infty }\log_{2}\left(1+x\right)f_{\Xi_{k}^{s}}\left(x\right)dx\overset{\left(a\right)}{=}\frac{1}{\ln\left(2\right)}\int_{0}^{\infty }\frac{1}{1+x}{\bar{F}}_{\Xi_{k}^{s}}\left(x\right)dx\\ \; & \overset{\left(b\right)}{=}\frac{1}{\ln\left(2\right)}\left(A_{k,c}^{s}\left(\prod_{i=1,i\neq k}^{N}B_{k,i}^{s}\right)\right)\int_{0}^{\infty }\frac{1}{1+x}\exp\left(-\frac{x\sigma_{k}^{2}}{P_{D_{k}}^{s}\Omega_{dr_{k},dt_{k}}}\right)\\ \; & \times \left(\frac{C_{k,c}^{s}}{A_{k,c}^{s}+x}+\sum_{i=1,i\neq k}^{N}\frac{D_{k,i}^{s}}{B_{k,i}^{s}+x}\right)dx\\ \; & =\frac{1}{\ln\left(2\right)}\left(A_{k,c}^{s}\left(\prod_{i=1,i\neq k}^{N}B_{k,i}^{s}\right)\right)\left(C_{k,c}^{s}H_{1}\left(\frac{\sigma_{k}^{2}}{P_{D_{k}}^{s}\Omega_{dr_{k},dt_{k}}},A_{k,c}^{s},1\right)\right.\\ \; & \left.+\sum_{i=1,i\neq k}^{N}D_{k,i}^{s}H_{1}\left(\frac{\sigma_{k}^{2}}{P_{D_{k}}^{s}\Omega_{dr_{k},dt_{k}}},B_{k,i}^{s},1\right)\right), \end{array}$$

where $\left(a\right)$ is obtained by applying the integral by parts and ${\bar{F}}_{\Xi_{k}^{s}}\left(x\right)$ is the complementary cumulative distribution function (CCDF) of random variable $\Xi_{k}^{s}$; and $\left(b\right)$ is held by using following identity

(14) $$\begin{array}{c} \prod_{i=1,}^{N}\frac{1}{x+\varphi_{i}}=\sum_{i=1}^{N}\frac{a_{i}}{x+\varphi_{i}};a_{i}={\left.\prod_{k=1,k\neq i}^{N}\frac{1}{x+\varphi_{k}}\right|}_{x=-\varphi_{i}} \end{array}$$

(15) $$\begin{array}{cc} H_{1}\left(a,b,c\right)= & \int_{0}^{\infty }\frac{1}{c+x}\frac{1}{b+x}\exp\left(-ax\right)dx=\frac{1}{c-b}\int_{0}^{\infty }\frac{\exp\left(-ax\right)}{b+x}dx+\frac{1}{b-c}\int_{0}^{\infty }\frac{\exp\left(-ax\right)}{c+x}dx\\ = & \frac{1}{c-b}\left[H_{2}\left(a,b\right)-H_{2}\left(a,c\right)\right]\\ H_{2}\left(a,b\right)= & \int_{0}^{\infty }\frac{\exp\left(-ax\right)}{b+x}dx\overset{\left(a\right)}{=}\exp\left(ab\right)E_{1}\left(ab\right), \end{array}$$

where $C_{k,c}^{s}={\left.\prod_{i=1,i\neq k}^{N}\frac{1}{B_{k,i}^{s}+\gamma }\right|}_{\gamma =-A_{k,c}^{s}}$, $D_{k,i}^{s}={\left.\frac{1}{A_{k,c}^{s}+\gamma }\prod_{u=1,u\neq k,i}^{N}\frac{1}{B_{k,u}^{s}+\gamma }\right|}_{\gamma =-B_{k,i}^{s}}$; $E_{1}\left(.\right)$ is the exponential integral function ([58], 8.211.1); and $\left(a\right)$ is obtained with the help of ([58], 3.352.4).

### 4.3. Average Sum Rate of D2D Networks

Having obtained the average rate of each D2D transmission, we now compute the average sum rate of the whole D2D networks under the *s* scheme (denoted by ${\bar{R}}_{\sum }^{s}$) as follows:

(16) $$\begin{array}{c} {\bar{R}}_{\sum }^{s}=\sum_{k=1}^{N}{\bar{R}}_{k}^{s}. \end{array}$$

### 4.4. The Amount of Fading (AoF) of D2D Link

The amount of fading is a fundamental metric to quantify the fading severity of a specific channel and is measured by the ratio of the variance to the square of the mean of the received signal. In particular, the AoF of the *k*-th D2D receiver of the $s\in \left\{equ,ran,pl\right\}$ scheme is given as [59]

(17) $$\begin{array}{cc} {\mathrm{AoF}}_{k}^{s}= & \frac{\mathrm{Var}\left\{\Xi_{k}^{s}\right\}}{{\left(E\left\{\Xi_{k}^{s}\right\}\right)}^{2}}\\ \mathrm{Var}\left\{\Xi_{k}^{s}\right\}= & E\left\{{\left(\Xi_{k}^{s}\right)}^{2}\right\}-{\left(E\left\{\Xi_{k}^{s}\right\}\right)}^{2}\\ E\left\{{\left(\Xi_{k}^{s}\right)}^{2}\right\}= & \left(A_{k,c}^{s}\left(\prod_{i=1,i\neq k}^{N}B_{k,i}^{s}\right)\right)\left(C_{k,c}^{s}H_{3}\left(\frac{\sigma_{k}^{2}}{P_{D_{k}}^{s}\Omega_{dr_{k},dt_{k}}},A_{k,c}^{s}\right)\right.\\ \; & \left.+\sum_{i=1,i\neq k}^{N}D_{k,i}^{s}H_{3}\left(\frac{\sigma_{k}^{2}}{P_{D_{k}}^{s}\Omega_{dr_{k},dt_{k}}},B_{k,i}^{s}\right)\right)\\ E\left\{\Xi_{k}\right\}= & \left(A_{k,c}^{s}\left(\prod_{i=1,i\neq k}^{N}B_{k,i}^{s}\right)\right)\left(C_{k,c}^{s}H_{2}\left(\frac{\sigma_{k}^{2}}{P_{D_{k}}^{s}\Omega_{dr_{k},dt_{k}}},A_{k,c}^{s}\right)\right.\\ \; & \left.+\sum_{i=1,i\neq k}^{N}D_{k,i}^{s}H_{2}\left(\frac{\sigma_{k}^{2}}{P_{D_{k}}^{s}\Omega_{dr_{k},dt_{k}}},B_{k,i}^{s}\right)\right), \end{array}$$

where $\mathrm{Var}\left\{X\right\}$ is the variance of RV *X*, $H_{3}\left(a,b\right)$ is defined as

(18) $$\begin{array}{cc} H_{3}\left(a,b\right)= & \int_{0}^{\infty }\frac{2x}{x+b}\exp\left(-ax\right)dx=2\left[\int_{0}^{\infty }\left(1-\frac{b}{x+b}\right)\exp\left(-ax\right)dx\right]\\ = & 2\left[\frac{1}{a}-H_{2}\left(a,b\right)\right]. \end{array}$$

**Proof.**  To prove (17), let us first compute the expectation of the e2e SINR as follows

(19) $$\begin{array}{cc} E\left\{\Xi_{k}^{s}\right\}= & \int_{0}^{\infty }\mathrm{Pr}\left\{X>x\right\}dx=\int_{0}^{\infty }{\bar{F}}_{X}\left(x\right)dx\\ \overset{\left(a\right)}{=} & \left(A_{k,c}^{s}\left(\prod_{i=1,i\neq k}^{N}B_{k,i}^{s}\right)\right)\int_{0}^{\infty }\exp\left(-\frac{x\sigma_{k}^{2}}{P_{D_{k}}^{s}\Omega_{dr_{k},dt_{k}}}\right)\\ \; & \times \left(\frac{C_{k,c}^{s}}{A_{k,c}^{s}+x}+\sum_{i=1,i\neq k}^{N}\frac{D_{k,i}^{s}}{B_{k,i}^{s}+x}\right)dx\\ = & \left(A_{k,c}^{s}\left(\prod_{i=1,i\neq k}^{N}B_{k,i}^{s}\right)\right)\left(C_{k,c}^{s}H_{2}\left(\frac{\sigma_{k}^{2}}{P_{D_{k}}^{s}\Omega_{dr_{k},dt_{k}}},A_{k,c}^{s}\right)\right.\\ \; & \left.+\sum_{i=1,i\neq k}^{N}D_{k,i}^{s}H_{2}\left(\frac{\sigma_{k}^{2}}{P_{D_{k}}^{s}\Omega_{dr_{k},dt_{k}}},B_{k,i}^{s}\right)\right), \end{array}$$

where $H_{2}\left(a,b\right)$ is provided (15). Next, we are going to compute the $E\left\{{\left(\Xi_{k}^{s}\right)}^{2}\right\}$ as follows

(20) $$\begin{array}{cc} E\left\{{\left(\Xi_{k}^{s}\right)}^{2}\right\}= & 2\int_{0}^{\infty }x\mathrm{Pr}\left\{X>x\right\}dx=2\int_{0}^{\infty }x{\bar{F}}_{X}\left(x\right)dx\\ \overset{\left(a\right)}{=} & 2\left(A_{k,c}^{s}\left(\prod_{i=1,i\neq k}^{N}B_{k,i}^{s}\right)\right)\int_{0}^{\infty }x\exp\left(-\frac{x\sigma_{k}^{2}}{P_{D_{k}}^{s}\Omega_{dr_{k},dt_{k}}}\right)\\ \; & \times \left(\frac{C_{k,c}^{s}}{A_{k,c}^{s}+x}+\sum_{i=1,i\neq k}^{N}\frac{D_{k,i}^{s}}{B_{k,i}^{s}+x}\right)dx\\ = & 2\left(A_{k,c}^{s}\left(\prod_{i=1,i\neq k}^{N}B_{k,i}^{s}\right)\right)\left(C_{k,c}^{s}H_{3}\left(\frac{\sigma_{k}^{2}}{P_{D_{k}}^{s}\Omega_{dr_{k},dt_{k}}},A_{k,c}^{s}\right)\right.\\ \; & \left.+\sum_{i=1,i\neq k}^{N}D_{k,i}^{s}H_{3}\left(\frac{\sigma_{k}^{2}}{P_{D_{k}}^{s}\Omega_{rk,tk}},B_{k,i}^{s}\right)\right). \end{array}$$

Combining (19) and (20), we obtain (17). Q.E.D. □

#### Coverage Probability of Cellular User

The coverage probability of cellular networks defines the probability that the cellular user is successfully served by the base station and is computed as follows [60]:

(21) $$\begin{array}{cc} F_{\Delta^{s}}\left(\gamma_{C}\right)= & \mathrm{Pr}\left(\Delta^{s}=\frac{P_{C}{\left|h_{b,c}\right|}^{2}}{\sum_{i=1}^{N}P_{D_{i}}^{s}{\left|h_{b,ti}\right|}^{2}+\sigma_{b}^{2}}\geq \gamma_{C}\right)=\mathrm{Pr}\left(\frac{P_{C}{\left|h_{b,c}\right|}^{2}}{I_{b}+\sigma_{b}^{2}}\geq \gamma_{C}\right)\\ = & \mathrm{Pr}\left({\left|h_{b,c}\right|}^{2}\geq \frac{\gamma_{C}}{P_{C}}\left(I_{b}+\sigma_{b}^{2}\right)\right)\\ = & \int_{i=0}^{\infty }\exp\left(-\frac{\gamma_{C}\sigma_{b}^{2}}{P_{C}\Omega_{b,c}}-\frac{\gamma_{C}}{P_{C}\Omega_{b,c}}i\right)f_{I_{b}}\left(i\right)di\\ = & \exp\left(-\frac{\gamma_{C}\sigma_{b}^{2}}{P_{C}\Omega_{b,c}}\right)\int_{i=0}^{\infty }\exp\left(-\frac{\gamma_{C}}{P_{C}\Omega_{b,c}}i\right)f_{I_{b}}\left(i\right)di\\ = & \exp\left(-\frac{\gamma_{C}\sigma_{b}^{2}}{P_{C}\Omega_{b,c}}\right)M_{I_{b}}\left(\frac{\gamma_{C}}{P_{C}\Omega_{b,c}}\right)\\ = & \exp\left(-\frac{\gamma_{C}\sigma_{b}^{2}}{P_{C}\Omega_{b,c}}\right)\left(\prod_{i=1}^{N}\frac{F_{b,i}^{s}}{F_{b,i}^{s}+\gamma_{C}}\right), \end{array}$$

where $F_{b,i}^{s}=\frac{P_{C}\Omega_{b,c}}{P_{D_{i}}^{s}\Omega_{b,ti}}$; $I_{b}=\sum_{i=1}^{N}P_{D_{i}}^{s}{\left|h_{b,ti}\right|}^{2}$ is the aggregate interference of D2D networks at the BS; and $\gamma_{C}=2^{R_{C}}-1$, $R_{C}$ is the target rate of the cellular user in bits/s/Hz.

### 4.5. Performance Trends

In this section, we investigate the impact of the total transmit power of the D2D networks on the performance of the outage probability. In particular, the behavior of OP with respect to $P_{\mathrm{tot}}$ is given in Proposition 1.

**Proposition** **1.** *The OP of $\Xi_{k}^{s}$, i.e., ${\mathrm{OP}}_{k}^{s}\left(\gamma \right)$ is monotonically decreasing with $P_{\mathrm{tot}}$.*

**Proof.**  Let us begin the proof by rewriting the OP as follows

(22) $$\begin{array}{cc} {\dot{\mathrm{OP}}}_{k}^{s}\left(\gamma ;P_{\mathrm{tot}}\right)= & \frac{d{\mathrm{OP}}_{k}^{s}\left(\gamma ;P_{\mathrm{tot}}\right)}{dP_{\mathrm{tot}}}=-\frac{\gamma \sigma_{k}^{2}}{{\left(P_{\mathrm{tot}}\right)}^{2}\omega_{k}^{s}\Omega_{dr_{k},dt_{k}}}\exp\left(-\frac{\gamma \sigma_{k}^{2}}{P_{D_{k}}^{s}\Omega_{dr_{k},dt_{k}}}\right)\frac{A_{k,c}^{s}}{A_{k,c}^{s}+\gamma }\\ \; & \times \left(\prod_{i=1,i\neq k}^{N}\frac{B_{k,i}^{s}}{B_{k,i}^{s}+\gamma }\right)-\frac{\omega_{k}^{s}\Omega_{dr_{k},dt_{k}}}{P_{C}\Omega_{dr_{k},c}}\exp\left(-\frac{\gamma \sigma_{k}^{2}}{P_{D_{k}}^{s}\Omega_{dr_{k},dt_{k}}}\right)\frac{\gamma }{{\left(A_{k,c}^{s}+\gamma \right)}^{2}}\\ \; & \times \left(\prod_{i=1,i\neq k}^{N}\frac{B_{k,i}^{s}}{B_{k,i}^{s}+\gamma }\right)<0, \end{array}$$

where ${\dot{F}}_{X}\left(x\right)=\frac{dF_{X}\left(x\right)}{dx}$ is the first-order derivative of *F* with respect to *x*. Q.E.D. □

## 5. Simulation Results

In this section, we present numerical results to verify the accuracy of the proposed mathematical framework. More precisely, we consider a generic cell with a circle shape, where the BS is located at the center and radius O=1000 m. Unless otherwise stated, the following parameters are employed throughout this section: N=4, BW = 1 MHz, η=3.6, $f_{c}=2.1$ GHz, NF = 6 dB, $P_{\mathrm{tot}}$ = 20 dBm, $P_{C}$ = 20 dBm, $R=R_{C}=1$ bits/s/Hz; and $\alpha =\left\{0.3650,0.2628,0.2700,0.1022\right\}$. The positions of D2D transmitter and D2D receiver are $P_{dt}=\left(-441,-92\right),\left(146,-33\right),\left(734,153\right),\left(-639,-633\right)$ and $P_{dr}=\left(-443,-95\right),\left(146,-39\right),$(740,148),(−646,−626), respectively, while the position of cellular user is (290,78).

**Figure 2:**
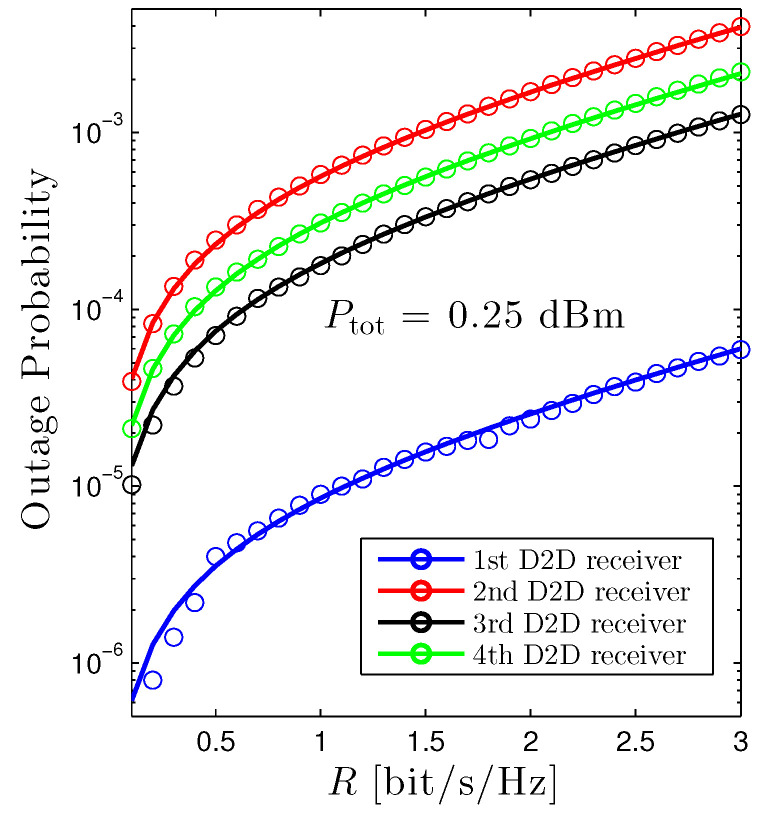
Outage probability of D2D receiver vs. *R* under the equal power allocation scheme. Solid lines are plotted by employing (12) and markers are Monte Carlo simulation.

Figure 2 illustrates the outage probability of all D2D receivers as a function of the target rate *R* under the equal power allocation scheme. Firstly, it is evident that the proposed mathematical framework absolutely matches with Monte Carlo simulations, confirming the accuracy of the mathematical frameworks. Secondly, under the current setup, the first D2D receiver achieves the best performance among all D2D receivers. It can be explained that it has the shortest transmission distance and is far away from the cellular user. On the other hand, despite benefiting from a moderate transmission distance, the performance of the second D2D receiver is the worst, owing to the largest interference from the cellular user. Thirdly, it is apparent that the higher the target rate, the worse the OP. The explanation is directly attained from the definition of the outage probability.

**Figure 3:**
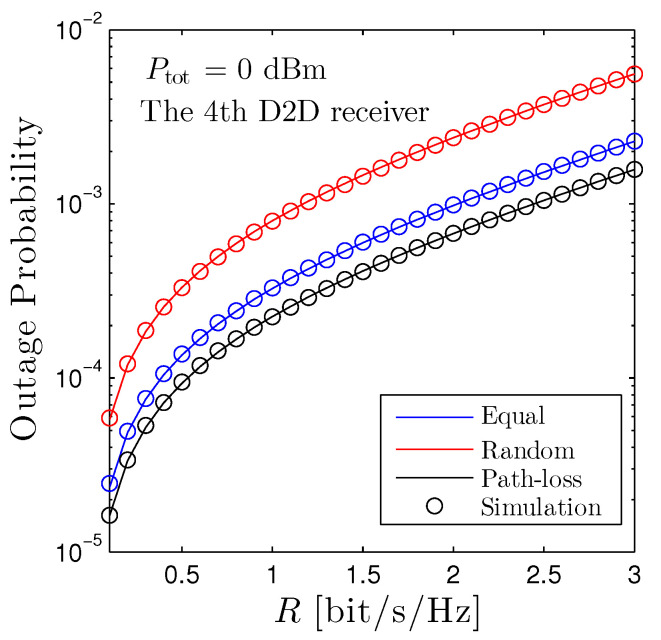
Outage probability of the fourth D2D receiver vs. *R* under all power allocation methods. Solid lines are plotted by employing (12) and markers are Monte Carlo simulation.

Figure 3 shows the outage probability of the fourth D2D receiver under all schemes with respect to the targeted rate. We observe that, under the current set up, the path-loss based scheme is the best one and the random scheme is, on the other hand, the worst one. More precisely, when R=1 [bits/s/Hz], the OP of the path-loss based scheme is approximately 0.0002, while the OP of the random scheme is 0.0008. This means that the path-loss based scheme is 4× better than its counterpart. However, all schemes are below 0.001, which means that the successful rate is 99.999%, securing the high-reliability transmission. Once again, the curves from the mathematical framework are perfectly overlapped with the computer-based simulation results.

**Figure 4:**
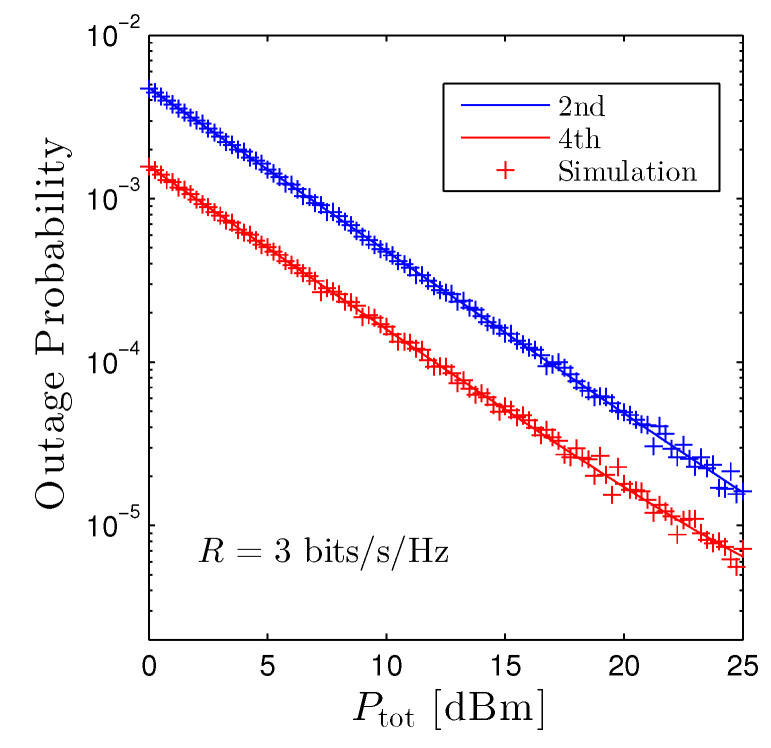
Outage probability vs. $P_{\mathrm{tot}}$ under the path-loss based method. Solid lines are plotted by employing (12) and markers are Monte Carlo simulation.

Figure 4 reveals the OP of the second and the fourth D2D receivers versus the total transmit power of the D2D networks under the path-loss based method. It is obvious that increasing the transmit power monotonically decreases the OP, hence confirming our findings in Proposition 1. The performance of the fourth receiver is better than the second receiver, as already shown in Figure 2. Nevertheless, the gap between two curves is moderate, and both schemes are below $10^{-4}$ when $P_{\mathrm{tot}}>20$ dBm.

**Figure 5:**
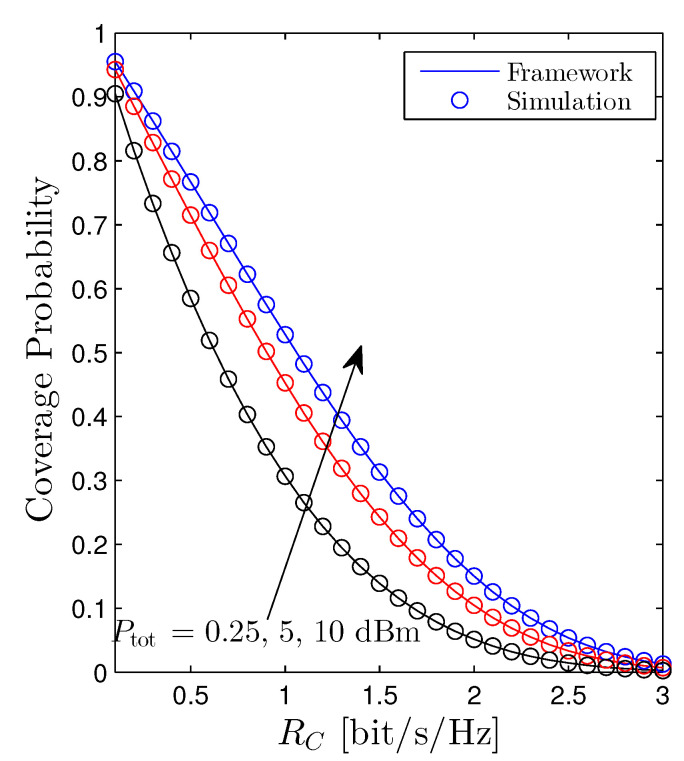
Coverage probability vs. $R_{C}$. Solid lines are plotted by employing (21) and markers are Monte Carlo simulation.

The coverage probability of the cellular user as a function of $R_{C}$ is shown in Figure 5. It is straightforward to recognize that raising the total transmit power of the D2D networks evidently degrades the performance of the cellular networks, e.g., the coverage probability goes down. Moreover, the Pcov simply declines with respect to the $R_{C}$ from the definition of the coverage probability.

**Figure 6:**
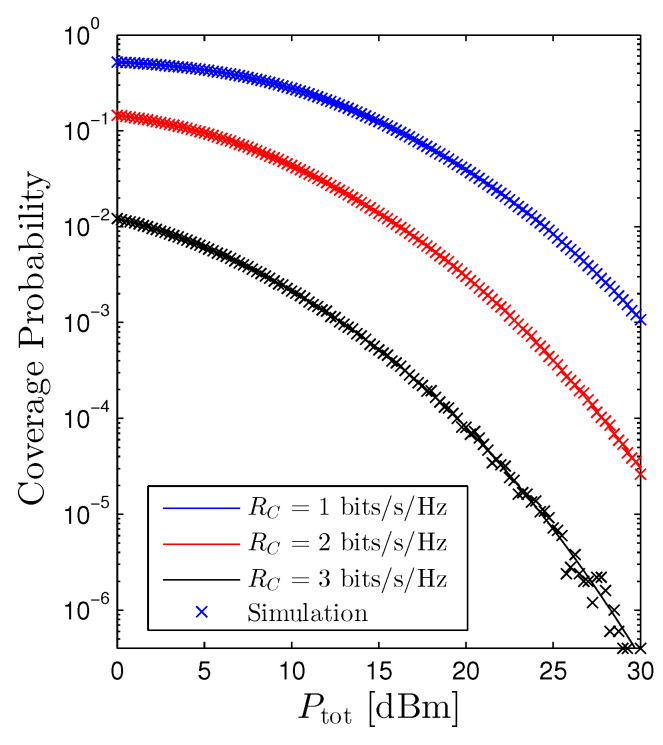
Coverage probability vs. $P_{\mathrm{tot}}$ under the random scheme. Solid lines are plotted by employing (21) and markers are Monte Carlo simulation.

Figure 6 illustrates the behavior of the Pcov versus the total transmit power $P_{\mathrm{tot}}$. We experience that increasing $P_{\mathrm{tot}}$ is harmful to the cellular networks. Hence, from Figure 4 and Figure 6, the impact of the $P_{\mathrm{tot}}$ on the performance of the OP and Pcov is contrary. In particular, increasing $P_{\mathrm{tot}}$ is beneficial for the D2D receiver. However, it is detrimental for the cellular user. As a consequence, figuring out an optimal total transmit power of the D2D networks that both guarantees the quality-of-service (QoS) of the cellular networks and ameliorates the performance of the D2D networks is an important extension direction. Figure 6 also confirms the findings in Figure 5 that scaling up the $R_{C}$ will reduce the Pcov.

**Figure 7:**
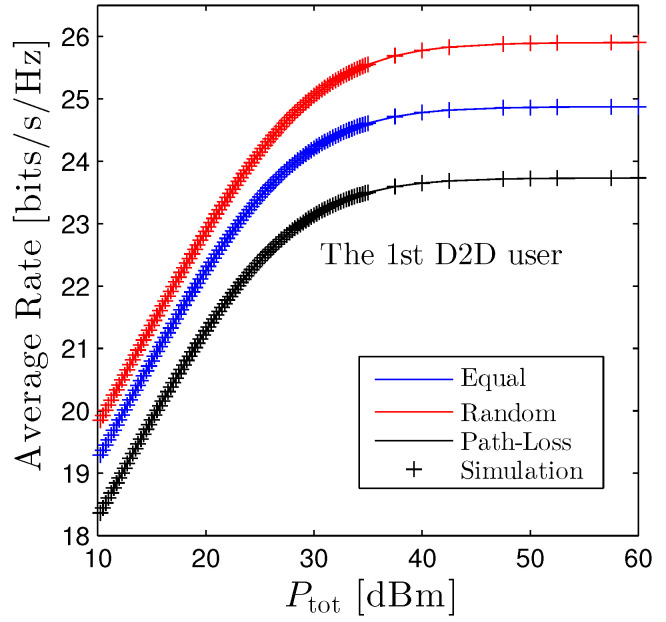
Average rate of the first D2D pairs vs. $P_{\mathrm{tot}}$ under three power allocation methods. Solid lines are plotted by employing (13) and markers are Monte Carlo simulation.

Figure 7 studies the performance of the average rate of the first D2D user, ${\bar{R}}_{1}$ versus $P_{\mathrm{tot}}$ under three power allocation schemes. We observe that, under the current setup, the ${\bar{R}}_{1}^{ran}$ outperforms the others. The rationale behind this phenomenon is that the allocated power of the random scheme is larger than ones utilized equal and path-loss based allocation, e.g., $\alpha_{1}>\frac{1}{N}>\frac{\Omega_{dr_{1},dt_{1}}}{\sum_{k=1}^{N}\Omega_{dr_{k},dt_{k}}}$. Nonetheless, the gap between these schemes is negligible, for example, ${\bar{R}}_{1}^{ran}$ is only better than ${\bar{R}}_{1}^{pl}$ by approximately 2 bits/s/Hz when $P_{\mathrm{tot}}=60$ dBm. Furthermore, the ${\bar{R}}_{1}$ is a monotonic increasing function with respect to $P_{\mathrm{tot}}$. Nevertheless, ${\bar{R}}_{1}$ linearly goes up as $P_{\mathrm{tot}}$ is small or moderate, e.g., when $P_{\mathrm{tot}}\leq 30$ dBm, then it fairly improves when $P_{\mathrm{tot}}>30$.

**Figure 8:**
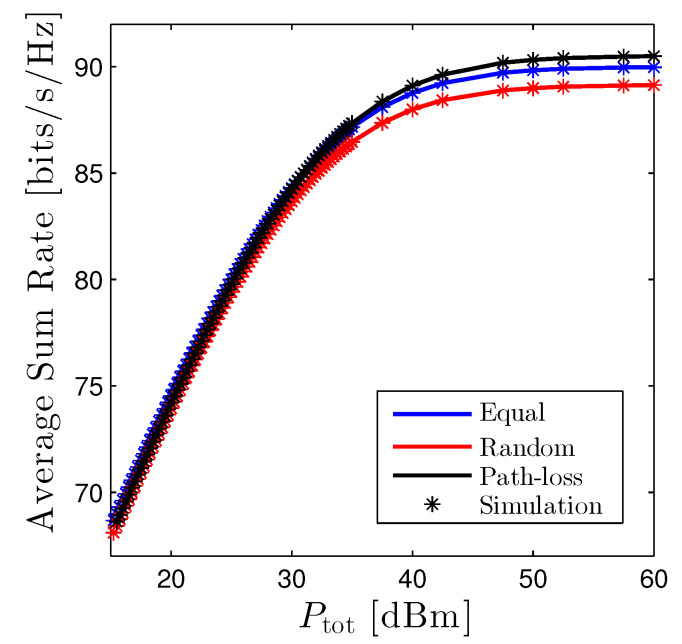
Average Sum Rate of D2D networks vs. $P_{\mathrm{tot}}$ under three power allocation methods. Solid lines are plotted by employing (16) and markers are Monte Carlo simulation.

The average sum rate of the D2D networks versus $P_{\mathrm{tot}}$ is given in Figure 8. Generally, the path-loss based scheme achieves the highest performance, although the difference between the best and the worst ones is minor, especially when $P_{\mathrm{tot}}$ is small. However, it is expected that the gap will be increased significantly providing that the number of D2D users is enormous.

**Figure 9:**
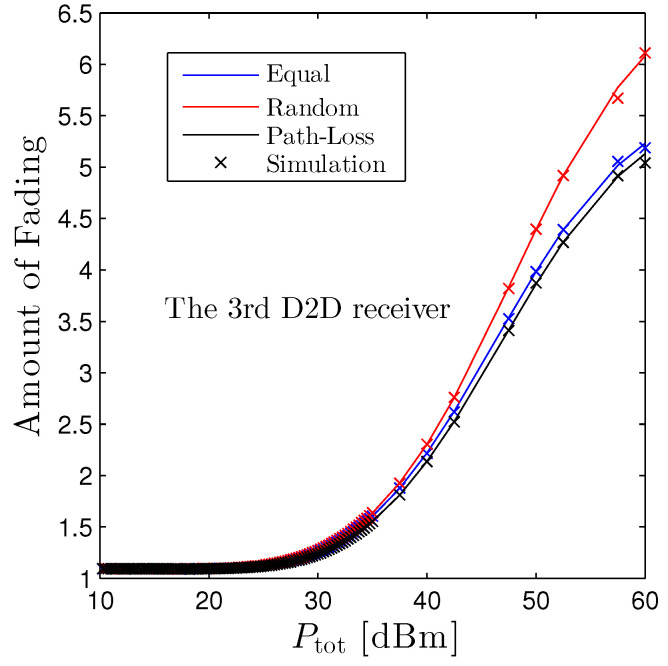
Amount of Fading vs. $P_{\mathrm{tot}}$ under three distinct schemes. Solid lines are plotted by employing (17) and markers are Monte Carlo simulation.

Figure 9 unveils the amount of fading of the third D2D receiver with respect to the $P_{\mathrm{tot}}$. We see that AoF simply boosts up with $P_{\mathrm{tot}}$, and the random scheme experiences the highest AoF while the path-loss based scheme is the lowest one for this receiver.

**Figure 10:**
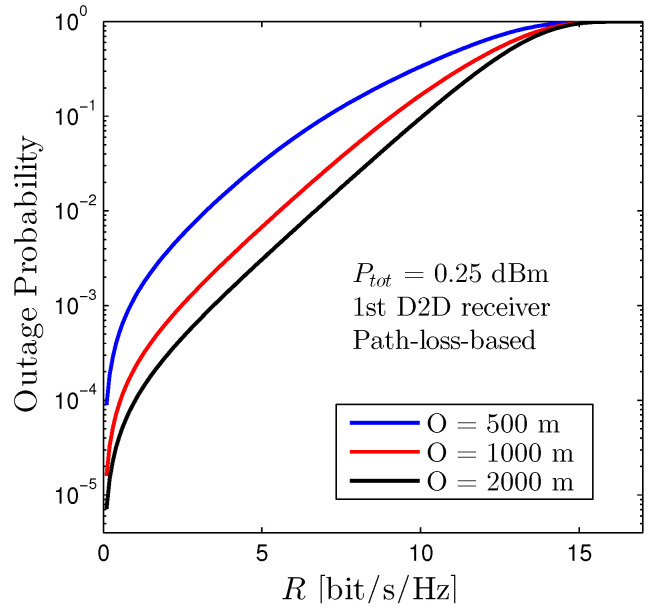
Outage Probability of the first D2D receiver vs. *R* under various values of network radius *O*. Solid lines are plotted by employing (12). The location of all devices (both D2D transmitters, receivers, and cellular users) are randomly distributed under the coverage area of the BS.

Figure 10 depicts the outage performance of the first D2D receiver with respect to *R* under the path-loss-based allocation scheme with various values of the network radius *O*. It is noted that the position of all devices (both D2D transmitters, receivers, and cellular users) in the considered networks is randomly distributed for each realization and the outcomes are drawn on an average of 1000 realizations. It is certain that increasing the network radius will ameliorate the outage performance. The rationale behind this trend is that increasing *O* will increase the distances between pairs of D2D transceivers and cellular users. As a result, the interference from both D2D and cellular networks declines.

**Figure 11:**
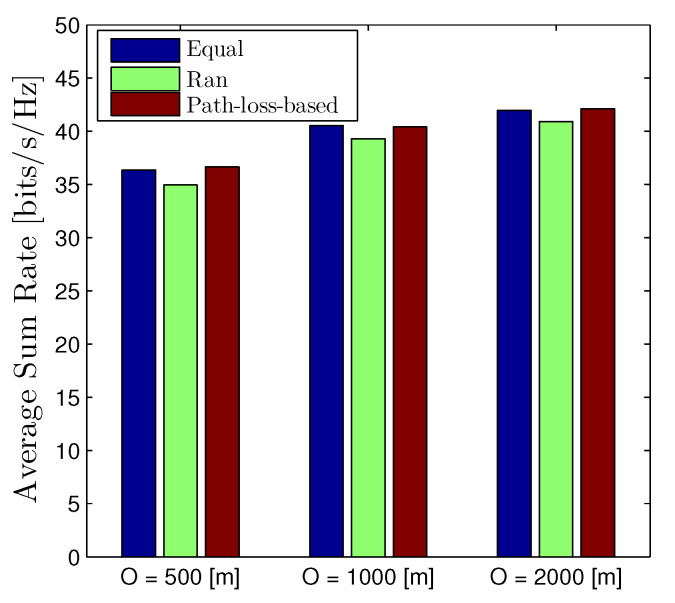
Average Sum Rate of D2D networks with different values of network radius *O*. The location of all devices (both D2D transmitters, receivers, and cellular users) are randomly distributed under the coverage area of the BS. Solid lines are plotted by employing (16).

Figure 11 stretches the average sum rate of the D2D networks with different network radius, i.e., O = 500, 1000, and 2000 m. Similarly, this figure is plotted by assuming that all nodes are randomly distributed under the coverage area of the BS for each realization. We observe that the behavior of the average sum rate with respect to the network radius *O* is similar to the outage probability that increasing *O* will improve the metric. Moreover, the path-loss-based scheme, again, is the best scheme, although the gap is minor, especially compared to the equal power allocation.

## 6. Conclusions

In this paper, we comprehensively investigated the performance of the single-cell underlay D2D networks. In particular, we derived the closed-form expressions of the outage probability, the average rate, and the amount of fading of D2D networks under three distinguished power allocation methods, i.e., the equal, random, and path-loss based schemes. Additionally, the coverage probability of the cellular user was provided. Moreover, we also presented the behavior of the OP with respect to the total transmit power of the D2D networks. Numerical results showed that the sum-rate under the path-loss-based scheme achieved the highest performance, followed by the equal, and the random scheme was the worst.

Additionally, our findings illustrated that the impact of the $P_{\mathrm{tot}}$ on the performance of the D2D and cellular networks are opposite. Thus, finding an optimal value of $P_{\mathrm{tot}}$ is an interesting potential for future work. Moreover, to further improve the system performance, multiple antennae at the transmitters and/or receivers can be adopted to exploit the diversity gain via diversity combining techniques [61,62,63,64]. The impact of the imperfect channel state information on the system performance is also valuable to address in future work [56,65,66,67,68]. Another promising extension of the present paper is to enhance the system performance of both the D2D and cellular networks with the support of the reconfigurable intelligent surface (RIS) [69,70,71] and/or utilizing interference alignment to mitigate mutual interference between two networks [72,73].

## Figures and Tables

**Table 1:** Main notations and mathematical symbols.

Symbol	Definition
$E\left\{.\right\}$, $\mathrm{Pr}\left(.\right)$	Expectation and probability operators
$h_{i,j}$	Channel coefficient between transmitter *i* and receiver *j*
$\Omega_{i,j}$	Large-scale path-loss between transmitter *i* and receiver *j*
$K_{0}$, $c_{0}$	Path-loss constant, speed of light
*v*, $f_{c}$, β	Wavelength, carrier frequency, path-loss exponent
$d_{i,j}$	Transmission distance from node *i* to node *j*
$P_{\mathrm{tot}}$	Total transmit power of D2D networks
$P_{D_{k}}^{s}$	Transmit power of the *k*-th D2D transmitter under *s* scheme
$P_{C}$	Transmit power of the cellular user
$I_{k}$	Aggregate interference at the *k*-th D2D receiver from D2D networks
$I_{b}$	Aggregate interference at the BS from D2D networks
$\alpha_{k}$	Weighted coefficient of the *k*-th D2D transmitter under random scheme
*N*, *O*	Number of pair of D2D users, network area
$x_{D_{k}}$, $x_{C}$	Transmitted signals of the *k*-th D2D transmitter and cellular user
$y_{D_{k}}$, $y_{C}$	Received signals at the *k*-th D2D receiver and base station
$n_{D_{k}}$, $n_{b}$	AWGN noise at the *k*-th D2D receiver and base station
$\sigma_{k}^{2}$, $\sigma_{b}^{2}$	Noise variance at the *k*-th D2D receiver and base station
NF, Bw	Noise figure, transmission bandwidth
R, $R_{C}$	Targeted rate of D2D networks and cellular networks
$\exp\left(.\right)$, $\ln\left(.\right)$	Exponential and logarithm functions
$E_{1}\left(.\right)$	Exponential Integral function
$F_{X}\left(x\right)$	Cumulative distribution function (CDF) of RV *X*
${\bar{F}}_{X}\left(x\right)$	Complementary Cumulative distribution function (CCDF) of RV *X*
$M_{X}\left(x\right)$	Moment generating function (MGF) of RV *X*
$f_{X}\left(x\right)$	Probability density function (PDF) of RV *X*
OP	Outage probability of the *k*-th D2D user under *s* allocation scheme
${\bar{R}}_{k}^{s}$	Average rate of the *k*-th D2D user under *s* allocation scheme
${\bar{R}}_{\sum }^{s}$	Average sum rate of the D2D networks under *s* allocation scheme
${\mathrm{AoF}}_{k}^{s}$	Amount of fading of the *k*-th D2D user under *s* allocation scheme
$\mathrm{Var}\left\{.\right\}$	Variance operator
$F_{\Delta }^{s}$	Coverage probability of cellular user under *s* allocation scheme
BER	Bit error rate
BS	Base station
D2D	Device-to-device
FDMA	Frequency-division multiple access
LoRa	Long-range
MIMO	Multiple-input multiple-output
NOMA	Non-orthogonal multiple access
QoS	Quality-of-service
RVs	Random variables
SINR	Signal-to-interference-plus-noise ratio
TDMA	Time-division multiple access
e2e	end-to-end

## References

[1] Yoon T., Nguyen T.H., Nguyen X.T., Yoo D., Jang B., Nguyen V.D. (2018). Resource Allocation for NOMA-Based D2D Systems Coexisting with Cellular Networks. IEEE Access.

[2] Hmila M., Fernández-Veiga M., Rodríguez-Pérez M., Herrería-Alonso S. (2021). Non-Orthogonal Multiple Access for Unicast and Multicast D2D: Channel Assignment, Power Allocation and Energy Efficiency. Sensors.

[3] Feng D., Lu L., Yuan-Wu Y., Li G.Y., Li S., Feng G. (2014). Device-to-device communications in cellular networks. IEEE Commun. Mag..

[4] Lee J., Lee J.H. (2019). Performance Analysis and Resource Allocation for Cooperative D2D Communication in Cellular Networks With Multiple D2D Pairs. IEEE Commun. Lett..

[5] Doppler K., Rinne M., Wijting C., Ribeiro C.B., Hugl K. (2009). Device-to-device communication as an underlay to LTE-advanced networks. IEEE Commun. Mag..

[6] Ghazanfari A., Björnson E., Larsson E.G. (2019). Optimized Power Control for Massive MIMO With Underlaid D2D Communications. IEEE Trans. Commun..

[7] Nguyen T.H., Chien T.V., Ngo H.Q., Tran X.N., Björnson E. (2021). Pilot Assignment for Joint Uplink-Downlink Spectral Efficiency Enhancement in Massive MIMO Systems With Spatial Correlation. IEEE Trans. Veh. Technol..

[8] He A., Wang L., Chen Y., Wong K., Elkashlan M. (2017). Spectral and Energy Efficiency of Uplink D2D Underlaid Massive MIMO Cellular Networks. IEEE Trans. Commun..

[9] Xu C., Feng J., Huang B., Zhou Z., Mumtaz S., Rodriguez J. (2017). Joint Relay Selection and Resource Allocation for Energy-Efficient D2D Cooperative Communications Using Matching Theory. Appl. Sci..

[10] Kamal M.S., Kader M.F., Islam S.M.R., Yu H. (2020). Device-to-Device Aided Cooperative Relaying Scheme Exploiting Spatial Modulation: An Interference Free Strategy. Sensors.

[11] Abro A., Deng Z., Memon K.A. (2019). A Lightweight Elliptic-Elgamal-Based Authentication Scheme for Secure Device-to-Device Communication. Future Internet.

[12] Xin J., Zhu Q., Liang G., Zhang T. (2020). Performance Analysis of D2D Communication with Retransmission Mechanism in Cellular Networks. Appl. Sci..

[13] Mustafa H.A., Shakir M.Z., Imran M.A., Imran A., Tafazolli R. (2015). Coverage Gain and Device-to-Device User Density: Stochastic Geometry Modeling and Analysis. IEEE Commun. Lett..

[14] Tu L.T., Di Renzo M. (2020). On the Energy Efficiency of Heterogeneous Cellular Networks With Renewable Energy Sources—A Stochastic Geometry Framework. IEEE Trans. Wirel. Commun..

[15] Qiao J., Shen X.S., Mark J.W., Shen Q., He Y., Lei L. (2015). Enabling device-to-device communications in millimeter-wave 5G cellular networks. IEEE Commun. Mag..

[16] Al Hajj M., Wang S., Thanh Tu L., Azzi S., Wiart J. (2020). A Statistical Estimation of 5G Massive MIMO Networks’ Exposure Using Stochastic Geometry in mmWave Bands. Appl. Sci..

[17] Tu L., Di Renzo M. Analysis of millimeter wave cellular networks with simultaneous wireless information and power transfer. Proceedings of the 2017 International Conference on Recent Advances in Signal Processing, Telecommunications & Computing (SigTelCom).

[18] Giatsoglou N., Ntontin K., Kartsakli E., Antonopoulos A., Verikoukis C. (2017). D2D-Aware Device Caching in mmWave-Cellular Networks. IEEE J. Sel. Areas Commun..

[19] Alves L.H.d.O., Rebelatto J.L., Souza R.D., Brante G. (2021). Network-Coded Cooperative LoRa Network with D2D Communication. IEEE Internet Things J..

[20] Tu L.T., Bradai A., Pousset Y. (2022). Coverage Probability and Spectral Efficiency Analysis of Multi-Gateway Downlink LoRa Networks. 10 Feb 2022. arXiv.

[21] Ming-Chun L., Molisch A.F., Ji M. (2021). Throughput-Outage Scaling Behaviors for Wireless Single-Hop D2D Caching Networks with Physical Model–Analysis and Derivations. arXiv.

[22] Amer A., Ahmad A.-M., Hoteit S. (2021). Resource Allocation for Downlink Full-Duplex Cooperative NOMA-Based Cellular System with Imperfect SI Cancellation and Underlaying D2D Communications. Sensors.

[23] Do D.-T., Nguyen M.-S.V., Lee B.M. (2019). Outage Performance Improvement by Selected User in D2D Transmission and Implementation of Cognitive Radio-Assisted NOMA. Sensors.

[24] Wang X., Cheng W., Xu X. On the Exact and Asymptotic Analysis of Wireless Transmission over *α* − *μ*/Inverse Gamma Composite Fading Channels. Proceedings of the 2020 International Conference on Wireless Communications and Signal Processing (WCSP).

[25] Cheng W., Wang X., Xu X. On the Performance Analysis of Wireless Transmission over *α* − *μ*/Inverse Gamma Composite Fading Channels. Proceedings of the 2020 IEEE/CIC International Conference on Communications in China (ICCC Workshops).

[26] Mankar P.D., Chen Z., Abd-Elmagid M.A., Pappas N., Dhillon H.S. (2021). Throughput and Age of Information in a Cellular-Based IoT Network. IEEE Trans. Wirel. Commun..

[27] Gorantla B.V.R., Mehta N.B. Subchannel Allocation with Low Computational and Signaling Complexity in 5G D2D Networks. Proceedings of the ICC 2021-IEEE International Conference on Communications.

[28] Cai Y., Ke C., Ni Y., Zhang J., Zhu H. (2021). Power allocation for NOMA in D2D relay communications. China Commun..

[29] Nguyen P.C., Rao B.D. (2015). Fair Scheduling Policies Exploiting Multiuser Diversity in Cellular Systems With Device-to-Device Communications. IEEE Trans. Wirel. Commun..

[30] Gu J., Bae S.J., Hasan S.F., Chung M.Y. (2016). Heuristic Algorithm for Proportional Fair Scheduling in D2D-Cellular Systems. IEEE Trans. Wirel. Commun..

[31] Fan B., Tian H., Jiang L., Vasilakos A.V. (2018). A Social-Aware Virtual MAC Protocol for Energy-Efficient D2D Communications Underlying Heterogeneous Cellular Networks. IEEE Trans. Veh. Technol..

[32] Saraereh O.A., Mohammed S.L., Khan I., Rabie K., Affess S. (2019). An Efficient Resource Allocation Algorithm for Device-To-Device Communications. Appl. Sci..

[33] Zhang L., Xiao M., Wu G., Li S. (2016). Efficient Scheduling and Power Allocation for D2D-Assisted Wireless Caching Networks. IEEE Trans. Commun..

[34] Lin Z., Song H., Pan D. (2019). A Joint Power and Channel Scheduling Scheme for Underlay D2D Communications in the Cellular Network. Sensors.

[35] Wang J., Song X., Ma Y. (2020). A Novel Resource Allocation Scheme in NOMA-Based Cellular Network with D2D Communications. Future Internet.

[36] Yuan H., Guo W., Wang S. Emergency route selection for D2D cellular communications during an urban terrorist attack. Proceedings of the 2014 IEEE International Conference on Communications Workshops (ICC).

[37] Yuan H., Guo W., Jin Y.L., Wang S., Ni M. (2017). Interference-Aware Multi-Hop Path Selection for Device-to-Device Communications in a Cellular Interference Environment. IET Commun..

[38] Chen G., Tang J., Coon J.P. (2018). Optimal Routing for Multihop Social-Based D2D Communications in the Internet of Things. IEEE Internet Things J..

[39] Singh I., Jaiswal R.K., Kumar V., Verma R., Singh N.P., Singh G. Outage Probability of Device-to-Device Communication Underlaying Cellular Network over Nakagami/Rayleigh Fading Channels. Proceedings of the 2019 9th International Conference on Emerging Trends in Engineering and Technology-Signal and Information Processing (ICETET-SIP-19).

[40] Jose J., Agarwal A., Gangopadhyay R., Debnath S. Outage Analysis based Channel Allocation for Underlay D2D Communication in Fading Scenarios. Proceedings of the 2019 International Conference on Wireless Communications Signal Processing and Networking (WiSPNET).

[41] Rodziewicz M. (2017). Outage Probability of Device-to-Device Communications in Frequency Reuse-1 Networks. Mob. Netw. Appl..

[42] Kusaladharma S., Zhang Z., Tellambura C. (2019). Interference and Outage Analysis of Random D2D Networks Underlaying Millimeter-Wave Cellular Networks. IEEE Trans. Commun..

[43] Hussain Z., Khan A.u.R., Mehdi H., Saleem S.M.A. (2018). Analysis of D2D Communications over Gamma/Nakagami Fading Channels. Eng. Technol. Appl. Sci. Res..

[44] Liu Y., Ding Z., Elkashlan M., Yuan J. (2016). Nonorthogonal Multiple Access in Large-Scale Underlay Cognitive Radio Networks. IEEE Trans. Veh. Technol..

[45] Renzo M.D., Zappone A., Lam T.T., Debbah M. (2018). System-Level Modeling and Optimization of the Energy Efficiency in Cellular Networks—A Stochastic Geometry Framework. IEEE Trans. Wirel. Commun..

[46] Thanh T.L., Renzo M.D., Coon J.P. MIMO cellular networks with Simultaneous Wireless Information and Power Transfer. Proceedings of the 2016 IEEE 17th International Workshop on Signal Processing Advances in Wireless Communications (SPAWC).

[47] Tu L.-T., Bradai A., Pousset Y., Aravanis A.I. (2021). Energy Efficiency Analysis of LoRa Networks. IEEE Wirel. Commun. Lett..

[48] Xia H., Li Y., Zhang H., Natarajan B. (2018). Availability of Ambient RF Energy in d-Dimensional Wireless Networks. Energies.

[49] Chien T.V., Björnson E., Larsson E.G. (2018). Joint Pilot Design and Uplink Power Allocation in Multi-Cell Massive MIMO Systems. IEEE Trans. Wirel. Commun..

[50] Nguyen T.N., Tran M., Nguyen T.-L., Ha D.-H., Voznak M. (2019). Multisource Power Splitting Energy Harvesting Relaying Network in Half-Duplex System over Block Rayleigh Fading Channel: System Performance Analysis. Electronics.

[51] Tin P.T., Phan V.-D., Nguyen T.N., Tu L.-T., Minh B.V., Voznak M., Fazio P. (2021). Outage Analysis of the Power Splitting Based Underlay Cooperative Cognitive Radio Networks. Sensors.

[52] Aravanis A.I., Tu L.-T., Muñoz O., Pascual-Iserte A., Di Renzo M. (2019). A tractable closed form approximation of the ergodic rate in Poisson cellular networks. EURASIP J. Wirel. Commun. Netw..

[53] Nguyen T.H., Jung W., Tu L.T., Chien T.V., Yoo D., Ro S. (2020). Performance Analysis and Optimization of the Coverage Probability in Dual Hop LoRa Networks With Different Fading Channels. IEEE Access.

[54] Chen Z., Ye C., Yuan J., Han D. (2019). MGF-Based Mutual Approximation of Hybrid Fading: Performance of Wireless/Power Line Relaying Communication for IoT. Sensors.

[55] Tin P.T., Nguyen T.N., Tran D.-H., Voznak M., Phan V.-D., Chatzinotas S. (2021). Performance Enhancement for Full-Duplex Relaying with Time-Switching-Based SWIPT in Wireless Sensors Networks. Sensors.

[56] Tu L.-T., Tung P.L., Chien T.V., Duy T.T., Hoa N.T. Performance Evaluation of Incremental Relaying in Underlay Cognitive Radio Networks with Imperfect CSI. Proceedings of the ICCE 2020.

[57] Nguyen T.N., Quang Minh T.H., Tran P.T., Vozňák M. (2018). Energy Harvesting over Rician Fading Channel: A Performance Analysis for Half-Duplex Bidirectional Sensor Networks under Hardware Impairments. Sensors.

[58] Gradshteyn I.S., Ryzhik I.M. (2007). Table of Integrals, Series, and Products.

[59] Lopez-Fernandez J., Lopez-Martinez F.J. (2020). New Results on the Second Order Scattering Fading Model: Amount of Fading and Energy Detection. IEEE Trans. Veh. Technol..

[60] Renzo M.D., Lam T.T., Zappone A., Debbah M. (2019). A Tractable Closed-Form Expression of the Coverage Probability in Poisson Cellular Networks. IEEE Wirel. Commun. Lett..

[61] Lam T.T., Renzo M.D., Coon J.P. (2016). System-level analysis of receiver diversity in SWIPT-enabled cellular networks. J. Commun. Netw..

[62] Thanh T.L., Renzo M.D., Coon J.P. Stochastic Geometry Analysis of Receiver Diversity in Cellular Networks with SWIPT. Proceedings of the 2018 IEEE 19th International Workshop on Signal Processing Advances in Wireless Communications (SPAWC).

[63] Minallah N., Ullah K., Frnda J., Cengiz K., Awais Javed M. (2021). Transmitter Diversity Gain Technique Aided Irregular Channel Coding for Mobile Video Transmission. Entropy.

[64] Do D.-T., Van Nguyen M.-S., Hoang T.-A., Lee B.M. (2019). Exploiting Joint Base Station Equipped Multiple Antenna and Full-Duplex D2D Users in Power Domain Division Based Multiple Access Networks. Sensors.

[65] Wang J., Song X., Ma Y., Xie Z. (2020). Power Efficient Secure Full-Duplex SWIPT Using NOMA and D2D with Imperfect CSI. Sensors.

[66] Chen Y., Zhang G., Xu H., Ren Y., Chen X., Li R. (2022). Outage Constrained Design in NOMA-Based D2D Offloading Systems. Electronics.

[67] Huang W., Han Z., Zhao L., Xu H., Li Z., Wang Z. (2021). Resource Allocation for Intelligent Reflecting Surfaces Assisted Federated Learning System with Imperfect CSI. Algorithms.

[68] Thanh T.L., Bao V.N.Q., An B. On the performance of outage probability in underlay cognitive radio with imperfect CSI. Proceedings of the 2013 International Conference on Advanced Technologies for Communications (ATC 2013).

[69] Chien T.V., Tu L.T., Chatzinotas S., Ottersten B. (2021). Coverage Probability and Ergodic Capacity of Intelligent Reflecting Surface-Enhanced Communication Systems. IEEE Commun. Lett..

[70] Chien T.V., Papazafeiropoulos A.K., Tu L.T., Chopra R., Chatzinotas S., Ottersten B. (2021). Outage Probability Analysis of IRS-Assisted Systems Under Spatially Correlated Channels. IEEE Wirel. Commun. Lett..

[71] Chien T.V., Tu L.T., Tran D.H., Nguyen H.V., Chatzinotas S., Renzo M.D., Ottersten B. Controlling smart propagation environments: Long-term versus short-term phase shift optimization. Proceedings of the IEEE ICASSP 2022.

[72] Cadambe V.R., Jafar S.A. (2008). Interference Alignment and Degrees of Freedom of the *K*-User Interference Channel. IEEE Trans. Inf. Theory.

[73] Song J., Tu L., Renzo M.D. On the feasibility of interference alignment in ultra-dense millimeter-wave cellular networks. Proceedings of the 2016 50th Asilomar Conference on Signals, Systems and Computers.

